# Lived experiences and challenges of older surgical patients during hospitalization for cancer: An ethnographic fieldwork

**DOI:** 10.3402/qhw.v9.22810

**Published:** 2014-02-13

**Authors:** Lisbeth Uhrenfeldt, Mette Terp Høybye

**Affiliations:** 1 Department of Public Health, Aarhus University, Aarhus, Denmark; 2 Department of Research, Horsens Hospital, Horsens, Denmark; 3 The Research Unit, Elective Surgery Center, Silkeborg Regional Hospital, Silkeborg, Denmark

**Keywords:** Lived experiences, caring, well-being, older patients, oral health, nutrition, colon cancer, ethnography

## Abstract

This paper explores the lived experiences of older surgical patients’ (aged 74 years and older) experienced challenges during a brief admission to hospital. Age, gender, polypharmacy, and the severity of illness are also factors known to affect the hospitalization process. For an ethnographic study using participant observation and interviews, surgical cancer patients (n = 9, aged 74 years and older) were recruited during admission to a Danish teaching hospital. Using ethnographic strategies of participant observation and interviews, each patient was followed through the course of 1 day during their stay at the hospital. Interviews were carried out with all patients during this time. Three areas of concern were identified as prominent in the patients’ experiences and challenges during their short hospital stay: teeth and oral cavity, eating in a hospital setting, and medication during hospitalization. Short-term hospitalization requires focused collaboration between staff and patient concerning individual challenges from their teeth and oral cavity as support of nutritional needs during surgical treatment for cancer.

One of the core challenges for old patients is poor oral health (Petersen & Yamamoto, 2005) and the need to consume substantial nutrition during their treatment and care (Brantervik, Jacobsson, Grimby, Wallen, & Bosaeus, 2005; Kondrup et al., 2002). Good oral health is essential to a continuous intake of nutrition (Heimdal, 1999; Öhrn, Sjöden, Wahlin, & Elf, 2001). Likewise, the oral health of older cancer patients has been found to be compromised during treatment in the hospital (Andersson, Persson, Rahm-Hallberg, & Renvert, 1999; Öhrn, Wahlin, & Sjödèn, 2001), a treatment in which patients are expected to be actively engaged (Andersson, Hallberg, Lorefalt, Unosson, & Renverts, 2004; Pedersen, 2005). Knowledge about older patients’ (aged 74 years and older) lived experiences of their oral health, however, is missing in the literature; therefore, this descriptive study is concerned with the lived experiences of older surgical patients and their daily challenges with intake of food and drinks during a brief admission to hospital.

Preparing this study included publishing of two literature reviews, one about adult patients’ perceptions of food and drinks while hospitalized (Larsen & Uhrenfeldt, 2013), and one on older surgical patients’ risk of malnutrition (Toppenberg & Uhrenfeldt, 2012). Both reviews point to the importance of the care setting and care practice on patients’ experience and intake of nutrients during hospitalization. The reviews represent the actual search strategy and literature outcome which is reported as background in this paper.

A discrepancy between the patient experience of meals in the hospital and the perceptions of healthcare professionals was found in the review, which documented the organization and environment of the hospital to affect the nutritional intake of patients (Larsen & Uhrenfeldt, 2013). Although patients left food because they were not able to eat without help, healthcare professionals believed that patients left food because they were full (Lassen, Kruse, & Bjerrum, 2005; Naithani, Whelan, Thomas, Gulliford, & Morgan, 2008). The importance of flavor, temperature, and texture of the served food and drinks; the significance of the eating environment; and the availability of food and drinks; affected their intake of nutrition during hospitalization (Larsen & Uhrenfeldt, 2013; Lassen et al., 2005; Sidenval, Fjellstrom, & Ek, 1994). In addition, age, gender, polypharmacy, the severity of illness, and the level of education were identified as factors influencing patients’ malnutrition (Toppenberg & Uhrenfeldt, 2012). Malnutrition is a problem among older patients in Europe (Brantervik et al., 2005; Pirlich et al., 2006; Vanderwee et al., 2010). Polypharmacy, an additional challenge to older patients’ well-being, identified in more severely ill patients as the intake of more than five different prescriptions per day (Pirlich et al., 2006), is commonly observed among geriatric patients (Stewart & Cooper, 1994). Among patients (mean age 65.9±17.7) with polypharmacy, unnecessary drug therapy, inappropriate choice of drugs, and untreated conditions, have been shown to prevail in hospitalized patients, with a reported incidence rate as high as 25% (Koh, Moideen, & Li, 2005). Many factors contribute to the high prevalence rate, but polypharmacy and old age have often been identified as important risk factors. The most common prescribed pharmacies (30–33% of the population investigated, n = 347) were a mild painkiller (paracetamol) and two laxatives (Senna and Lactulose). Nearly 10% of the study population had at least one drug-related problem at admission (Koh et al., 2005).

Furthermore, older patients with visual impairment (VI) are more than twice as likely to need help managing medication (McCann et al., 2012). The prevalence of VI is increasing, contributing to increased morbidity, particularly among older people. Despite the use of optical aids, few patients with VI in a previous study could read medication information clearly (McCann et al., 2012). One of four had difficulty distinguishing different tablets, and almost one in three needed help managing their medication from friends or pharmacists (McCann et al., 2012).

During a hospital stay, a number of central events occur such as admission, transfer between wards, and preparation for discharge (Coughlin, 2013). Such physical and psychological changes have been shown to affect the intake of nutrition in patients (Hallström, Elander, & Rooke, 2000; Larsson, Hedelin, & Athlin, 2003).

The support of nurses or next of kin through transitions has been found to influence patients’ experience of care and their confidence in the ability to get support (Uhrenfeldt et al., 2013). The interaction between nurses, patients, and next of kin through these changes also affect patients’ nutritional care (Holst, Rasmussen, & Unosson, 2009). The literature mainly informs us about older medical patients’ vulnerable situations (Holst, Mortensen, Jacobsen, & Rasmussen, 2010; Holst, Rasmussen, & Laursen, 2010; Wright, Hickson, & Frost, 2006); however, it excludes the specific problems older people experiences when their treatment for cancer is through a surgical fast-track program and their hospitalization mainly focuses on a surgical outcome. Likewise, due to new operating techniques and anesthesia, older people without age limit receive surgical treatment, but their lived experiences may differ due to personal life circumstances.

Qualitative research, and the ethnographic approach in particular, have the ability to explore and elucidate the situated and contextual differences of the individual experience of surgical treatment to open up a deeper awareness of the complexity. In this particular study, the ethnographic approach made it possible to draw on the lived experience of hospitalization due to cancer when dealing with the older surgical patient. Such engagement is dependent on the researcher being present in the field over a period of time to open the production of knowledge through observations and dialogue. Through this particular form of knowledge production, a deeper understanding of the complexity of the lived experience of surgery and care in older patients is obtained to complement the clinical data and reasoning around these patients.

## Aim of study

This paper explores older surgical patients’ (aged 74 years and older) experienced challenges during a brief admission to hospital.

## Methods

This qualitative study (Kvale & Brinkmann, 2009) is based on a hermeneutic understanding (Gadamer, 2004), in the sense of searching for truth in a co-creation between the researcher and the person being researched. Meaning occurs through a circle of reflective writings and interpretation between the whole and parts of the research and between pre-understanding and understanding (Alvesson & Sköldberg, 1994).

## Study design and setting

The study was conducted as a 3-month (March 2011–June 2011) ethnographic study (Hammersley & Atkinson, 2007) using participant observation, interviews, and reading of various clinical documents at a Danish teaching hospital. The hospital received patients from a geographical area of 220,000 inhabitants. The actual surgical ward had 44 beds, and the average length of stay in 2012 from January to July was 4.12 days and occupancy was 109.7%.

The ethnographic approach (Hammersley & Atkinson, 2007) was chosen in order to explore and open up the experience of nutritional intake during admission at a hospital among older surgical patients. The ethnographic research design used methods of participant observation recorded through field notes (Emerson, Fretz, & Shaw, 1995; Hammersley & Atkinson, 2007) that described the observations and interactions in the setting of the time spent with the patient and further reflective notes on the courses of the interaction. Using participant observation provided an opportunity to spend time with the patients over a period of time (1 day during admission), to observe the choices made and reach a deeper understanding of their experience of personal consumption of food and drinks during their admission and the practices surrounding the decisions of and intake of these. This method of research has previously been used successfully to reach an understanding of the sparsely verbalized and subdued clinical practices (e.g., Høybye, 2013; Mattingly, 1998).

The inclusion criteria were inspired by the initial literature search (Box 1). Based on the inclusion criteria, older patients of both genders were recruited by the ward sister. She contacted one or two patients the day before or on the morning of a research day and provided both oral and written information about the study. Patients were asked to provide written consent if they agreed to participate. When a patient consented to participate in the study, the ward sister contacted the researcher who arrived at the ward before breakfast was served.

**Box 1. Inclusion criteria**

Included participants:

In surgical treatment

Mentally present

Aged 74 years and older

>5 medications per day

Within first week of hospital stay

The term “older people” is not easily defined (Cheek, 2010); in this study we invited patients aged 74 years and older. Formal retirement age in Denmark is 65 years, which means that the surgical patients invited to participate in this study had been eligible for retirement from the labor market for approximately 10 years or more.

Patients’ medical records on diagnosis, prescribed medicine, and nutrition, including nurses’ objectives for the actual nursing care, were provided by the ward sister for the researcher's use. The medical records were used to determine the level of polypharmacy in each patient and to get a sense of their medical and surgical history during the present admission. The researcher anonymized these clinical documents by giving the patients a study participant number from 1–10 when received. The same participant numbers were used for field notes and as references throughout this paper. The text analysis of these documents revealed insights (Tables I and III) about main events during the individual patient's hospitalization (Coughlin, 2013).

**Table I:** Participants’ personal data and time of observation.

Participant	Age/gender/marital status	Time of observation
1	81/F/M	8.15–20.00 (12 h)
2	75/M/M	8.15–18.15 (10 h)
3	85/M/M	8.15–18.30 (10 h)
4	85/F/M	8.20–14.30 (6 h)
5	82/F/W	8.00–15.30 (7.5 h)
6	78/M/W	8.00–15.30 (7.5 h)
7	79/F/UM	8.00–12.45 (5 h)
8	74/M/M	7.45–13.00 (5 h)
9	75/F/W	8.00–15.00 (8 h)
	Age span: 74–85	Time: 5–12 h

### Participants

Ten older surgical patients were invited to participate in the study. All patients initially accepted participation but one withdrew her consent when she was approached by the researcher as she felt too tired to “be part of any of this” this day or any other day.

The patients included (n = 9) in the study were between 74 and 84 years of age. Participants were comprised of four men and five women, of whom five were still married, three were widowers, and one was unmarried. They were all ethnically Danish and had the diagnosis: cancer coli. They spent from 5 to 12 h with the researcher during 1 day of their brief admission to hospital.

### Collection of data

Participant observation (Table I) was carried out on weekdays by one researcher (LU) from 7:30 am until 8:30 pm with breaks depending on the endurance of the individual patient and the need for rest or privacy with personal visitors. The researcher arrived in the early morning while the patient was still in bed and followed the patient through his or her daily routine. In the morning, this meant following along with the patient to the dining room, or sitting at the bedside while breakfast was served, depending on each patient's situation. Later in the day, the dining room was revisited during lunch, afternoon coffee time, and in two cases for late supper and coffee as well. In between the planned meals, some patients were served or fetched drinks themselves. The researcher walked from bedside to dining room accompanying some patients who had gone in search of drinks or supplementary food.

Following selected patients by participant observation provided a possibility to understand the transitions and changes experienced by the patients due to their cancer and the hospital setting. The guiding element in the participant observation conducted in this study was observation (Holy, 1984), with participation in the clinical context serving as a means to be able to observe. Combining participant observation with interviews, as is commonly done (Hammersley & Atkinson, 2007), further strengthened the possibility to gain insight into the more subtle relationship between hospital admission and nutrition in older surgical patients with cancer coli or rectum.

The interviews were intertwined parts of the participant observations, both as semi-structured interviews, and as informal conversations (Kvale & Brinkmann, 2009). A guide (four main topics: daily habits, personal preferences, hospitalization, and oral health) (See Box 2) was created for the semi-structured interviews conducted with each patient during the day they were observed. The semi-structured interview was conducted in a separate room in the ward specifically used for private conversations (n = 6) if permitted by their general health condition (i.e., walking distance, oxygen mask, dizziness). Some patients (n = 3) were not able to leave their room. The interviews and informal conversations instead served a similar purpose, as has been argued by Rubow (2003), as sites of reflection that further provided access to understanding the experience of patients in the ethnographic field.

**Box 2. Semi-structured interview guide: Examples of questions in four main areas**

*Daily habits*

How would you describe your good habits concerning meals at home?

*Personal preferences*

What do you eat and drink in the ward, do you miss anything?

Are factors such as radio and television significant in relation to your intake of food and drink when you are at home?

Are radio and television significant in relation to your intake of food and drink in the ward?

*Hospitalization*

How does the staff help as far as your intake of food and drink in the ward?

What significance do fellow patients’ accounts have in relation to your eating and drinking?

Do you see any obstacles as far as your desire to drink and eat here?

*Oral health*

How is your mouth at the present moment, i.e., number of teeth, missing teeth, gums, impact on meals?

Shorthand field notes recorded observations and responses to questions. Influenced by practical ethnographic principles (Hammersley & Atkinson, 2007), paraphrased summaries were developed immediately after the interviews were completed. This is a suitable method in nursing research where seeing the big picture is a challenge due to the multiple and simultaneous actions and events in clinical practice (Bundgaard, Nielsen, Delmar, & Sørensen, 2012; Sørensen & Hall, 2011). All field notes and participants’ stories were transcribed on the day of or the day following the participant observation in order to capture the experience as accurately and nuanced as possible.

### Analysis of data

The analysis identified repeating and deviating patterns in the material to develop sensitizing concepts that, through the process of analysis, became more definitive concepts (Emerson et al., 1995), to draw out explanations for establishing knowledge based on older surgical patients’ wishes and preferences for food and drink while hospitalized. The analysis was discussed throughout the process between the two authors, representing two disciplines: nursing (LU) and anthropology (MTH).

The first step of analysis was to produce a brief overview of the sample by organizing data (Hammersley & Atkinson, 2007), presented as a summary (Tables II and III), and corresponding to the study aim. Second, all notes from observations and interviews were transcribed into a coherent text piece representing each participant. Third, these were read individually and together to identify similarities and differences in the participants’ stories and experiences (Miles & Huberman, 1994). Fourth, themes were developed, scrutinized, and refined through movements between the text parts and the texts as a whole, developing three sensitizing themes (Emerson et al., 1995): teeth and the oral cavity, eating in a hospital setting, and medication during hospitalization, from which the overall description of the relations of the experience of older surgical patients with polypharmacy during hospitalization and their choices and intake of food and drinks emerged. The analysis was conducted manually through the use of a word program (Microsoft 2003); first, through a naïve reading bringing forward meaning units, then, these meaning units were structured into sub-themes. During this process, a hermeneutical back and forth engagement completed the picture of each person investigated while reading and understanding each of the meaning units challenging the researchers pre-understanding and bringing forward new meaning units. This process resulted in sub-themes leading to core themes.

### Ethical considerations

The Central Denmark Regional Research council was notified about the study and the study protocol was registered (Data Protection Agency, journal no. 2007-58-0010).

Prior to initiating the study, its purpose and design was presented at a ward staff meeting. For patient information a handout was produced containing a short informative text on the study aim, actions, and precautions to ensure patient anonymity. The handout was presented to patients by the ward sister who selected and contacted the patients. The interviewer was alert to possible signs of non-predicted distress among the patients caused by the research process throughout the field observations, dialogues, and interviews (Kavanaugh & Ayres, 1998).

## The findings

The results are presented within three core themes emerging from the analysis: teeth and the oral cavity, eating in a hospital setting, and medication during hospitalization.

### Teeth and the oral cavity

The oral conditions and care of patients challenged their well-being. Exploring how this affected their consumption of food and drinks in the hospital, we found that three older persons had a full set of teeth while five were missing their molars, and one was missing her full set of teeth (Table II). The dental status of each patient was investigated during the interviews. There was some delay in replying to the question of the researcher on their individual dental status, a delay interpreted as unpreparedness for the topic as some patients replied that this was the first time anybody questioned this part of their body and, in some cases, they felt a sense of embarrassment concerning their dental status. The six informants with different missing teeth needed help with oral care due to either fungal attack or sore gums. The one patient who lacked all her teeth had an oral cavity contracture that made it painful for her to open and close her mouth, thus she preferred to sip a liquid diet and consequently was losing weight (Table II).

**Table II:** Participants’ surgical and nutritional situation.

Participant no./age	Diagnosis/operation	Nutritional assessment	Prescribed nutrition	Teeth status
1/81	Cancer rectum/	Nutrition risk/weighed twice weekly	All	Upper dental plate
2/75	Cancer coli sigmoideum/	Normal/stable	All	Missing molars in upper and lower part
3/85	Cancer coli/hemicolectomy	Normal/stable	All	Broken upper dental plate in front No molars at all in upper and lower part
4/85	Cancer coli/	Normal/stable	Fluid	2 missing molars in the upper part of mouth
5/82	Cancer coli/	Normal/stable	All	Full set of teeth
6/78	Cancer coli/colestomy	Nutrition risk/weighed twice weekly	All	Missing molars in upper and lower part
7/79	Obs cancer coli and pain in stomach/no treatment	Nutrition risk/weighed twice weekly	Fluid	No teeth at all
8/74	Cancer coli/hemicolectomy	Normal/stable	All	Full set of teeth
9/75	Cancer coli/	Normal/stable	All	Full set of teeth

A patient with a denture in the mouth described how it had fallen out while brushing his teeth in the hospital. It hit the floor and split in two. The patient described having picked it up, washed it and put it back in his mouth. The broken denture had produced bruising to his mouth causing him to eat only liquid or very soft food. He did not reflect further on the situation at the time or during the interview.

Another patient had a large gap between his incisors and lacked all his molars. Additionally, he suffered from thrush with swollen and dry membranes of the mouth. This made it very troublesome for him to eat anything, and he was further afflicted by his colostomy surgery. During the day the researcher spent with him, he was served a meal of boiled beef, light sauce, and potatoes for lunch. After the meal, his mouth clearly bothered him. He rinsed his mouth with water and started to gore his teeth with a fork, resulting in bits of meat hovering in all directions outside and on the bed. His facial expressions and breathing showed that he was bothered by the discomfort the bits of meat had left in his mouth.

The condition of their teeth, such as missing molars or a sore mouth due to factors such as a dental plate that did not fit properly after weight loss, resulted in difficulty chewing for some patients. This profoundly affected the experience and possibility of consuming food or drink during hospitalization. However, patients described how they experienced a continuous pressure from the professionals to increase their consumption of regular drinks as well as of protein-enhanced drinks.

### Eating in a hospital setting

Patients were admitted to hospital on the basis of a suspected or manifest diagnosis of cancer of the stomach or intestine. Before admission, they had often experienced a period of poor appetite, nausea, or insomnia due to indefinable pain. Admission was described by patients as a lonely experience offering stories of busy, adult children who often worked a lot or maybe lived far away and “were not to be burdened”, as some patients expressed it. Often older spouses were not capable of transporting themselves to the hospital for visits or participation in consultations with physicians and therefore, contact was primarily sustained only by telephone.

After undergoing gastrointestinal surgery, patients experienced fatigue, nausea, and pain in the operation wound and found it hard to get out of bed and walk around and to start eating again immediately after surgery. Despite this, some patients reflected that the nursing staff did not show sympathy to their experience and urged them to get moving and exercise by, for example, walking to the dining room, regardless of how hard it was for the patients to sit and to walk around.

Each dining room in the ward had five tables with seating for four people, which offered an opportunity to chat with fellow patients over a meal. However, there was also a loud television, which some patients perceived as an annoying disturbance during the meal. A long row of windows along one side of the dining room provided a view of a marina and open water, which the ambulatory patients described as a pleasant place to sit and eat with fellow patients.

Meals at the hospital were in several ways experienced as different from home by the participants. The written menu did not always arouse their interest in food, but after a visit to the buffet, they changed their minds. The smell and the sight of food awoke more interest and appetite. Conversations with the kitchen staff helped invoke the desire to eat, however the hunger did not always match the desire. Seven patients took in full nutrition while two patients only took in fluid nutrition (Table II). A patient with no appetite in one case had her appetite aroused when it was confirmed at the buffet that they were serving an old, traditional Danish dish “Mannagrød” (Manna porridge). This familiar food gave rise to a wide smile and comments of surprise and pleasure.

The lack of appetite was one of the challenges experienced. A woman expressed that the food at the hospital presented too much meat, was too salty, and did not contain enough vegetables; she said that her 92-year-old husband grew vegetables in an amount that made it possible for him to sell the extra crop during the summer. She said this with such pride. Her head lifted and she got a clear look in her eyes. This kind of recognizable, local and “easy chewable food”, however, was not served in the hospital, she claimed. Verbal interaction with the other patients was difficult to this woman, due to impaired hearing on both ears. This clearly isolated her in relation to others in the ward and added to her feeling miserable and homesick during her admission.

### Medication during hospitalization

From the individual records, information on the medication and condition of each patient was retrieved. Table III shows the difference in the medication of each patient, with an intake of 6–15 different medications. All patients received one to three nutritional supplements. Almost everyone (n = 8) got one to two weak analgesic medications, most patients (n = 7) got one to two blood clot inhibiting medications, and many received (n = 7) one to two medications for high blood pressure. More than half (n = 6) were treated with constipation preventive agents and (n = 5) with one or two strong painkillers. Other medical treatment allocation (infections, heart insufficiency, diuretics, elevated cholesterol, anxiety, arthritis, asthma, or nausea) was also present in the patients’ treatment (Table III).

**Table III:** Participants’ individual needs for medical treatment.

No./Med.	Nutritional supplement	Weak pain killers	Prevent blood clots	Prevent high blood pressure	Prevent constipation	Strong pain killers	Treat infection	Other**
1/9	v*	**v**	**vv**	**v**	**v**	**v**		vv
2/15	**vvv**	**v**	**vv**	**vv**		**v**		vvvvvv
3/9	**vv**	**v**	**v**	**v**	**v**		v	vv
4/5	**v**		**v**	**v**	**v**			v
5/8	**v**	**v**	**vv**	**vv**				vv
6/8	**v**	**v**			**v**	**vv**	vv	v
7/9	**v**	**vv**	**v**	**v**	**v**	**v**	v	v
8/7	**vvv**	**v**		**v**	**v**	**v**		
9/7	**vv**	**v**	**v**				vvv	
	**9 of 9**	**8 of 9**	**7 of 9**	**7 of 9**	**6 of 9**	**5 of 9**	4 of 9	2 of 9

Bold to illustrate the most dominant groups of treatment.

* v marks one medication.

** Medication received for treatment of either heart failure/diuretic, high cholesterol, acidity of stomach, anxiety, arthritis, adrenal cortex, asthma, or nausea.

The individual medication was delivered by a nurse and placed in a cup on the bedside table of each patient, unless the patient reached out and received the tablets directly in their hand. Then began the individual consumption of 6–15 different tablets. Some patients asked for yoghurt for breakfast for the sake of consuming up to 15 tablets, which did ease the procedure. Others took tablets with protein drinks. None of the participants complained about the amount of medication. Only one male stated (shrugging) that before he was admitted to the hospital, he had never once taken medication.

Field observations uncovered older trembling hands that had lost their precision grip but were still trying to capture the tablets by shaking them out by hand (and then losing some) or trying to pry one or two tablets out of the cup at a time with one or two fingers. The impaired vision of some patients made the whole operation rather uncertain. During an observation session with one of the male patients, he lost two of his many tablets on the floor during this procedure. He did however not discover the loss. For a couple of patients with impaired hearing, irrespective of using a hearing aid, it was difficult for an assistant to work with them on the consumption of medicine.

More patients reported to suffer from thrush. While observing a nurse performing oral care on one of the patients, a jammed tablet palate emerged from the day before. Its substance could not be determined and the patient seemed somewhat surprised by this too, since he had no sensation of a dry tablet in his mouth.

In summary, based on patients′ experiences, there are specific needs in the care for older cancer patients’ oral health and care for their consumption of food, drinks, and medication during their stay and recovery in a surgical hospital environment to support their well-being and meet the challenges facing their health situation.

## Discussions

This study describes situations experienced by older surgical cancer patients during their brief admission to hospital. Their experiences concerning the intake of food and drinks during hospitalization were a specific area of interest. Beyond the challenges described concerning hospital admission (Andersson et al., 2004; Kondrup et al., 2002; Pedersen, 2005) and a serious illness (Uhrenfeldt et al., 2013), a brief stay in a surgical ward can be further problematic due to oral health conditions of the older patient, such as sore oral cavity and high level of tooth loss, or when there is need for a specific intake of drinks and food to counteract malnutrition.

In our study, six of nine patients had missing molars and their experience demonstrates how this oral health challenge served as a hindrance for taking in sufficient nutrition and for their general well-being during their hospital stay. A previous study suggested that patients experienced feeling hungry during their hospital stay. Menus did not enable informed decisions concerning the food in order to meet specific nutritional needs and there were physical barriers to getting sufficient nutrition such as not being in a comfortable position to eat; however, there is no information about the discomfort from the oral cavity (Naithani et al. 2008; Toppenberg & Uhrenfeldt 2012). Our study adds new knowledge about the old patients’ oral cavity as a challenge to the nutritional balance and the significance of oral health for the ability of the individual patient to consume sufficient food and drink. The value of prioritizing serving in-between meals in a medical ward (Holst, Mortensen, et al., 2010) may also need more attention in surgical wards. Early evidence based on informed interactions with a specific focus on the prevention of malnutrition, may be a way to support the old surgical patient with cancer during a brief stay in hospital. A continuous focus on preventing malnutrition during the transition from hospital to home may require additional transfer of information between sectors on the patient's oral health status and nutritional preferences in collaboration with the individual patient.

Access to familiar food was an example of how one woman with no appetite had her appetite aroused and thereby experienced a bodily feeling of comfort that shaped a sense of “feeling at home” while enjoying her “Mannagrød” and forming a moment of dwelling and well-being. Drawing on the thoughts of dwelling and well-being by Galvin and Todres in 2011 of a movement between points followed by the ability to rest; the familiarity of the food served to this patient elicited well-being and produced a social connection enabling her to be able to enjoy her meal with others in the dining room and dwell in this moment, despite being seriously ill and admitted to hospital.

Remembering her husband's daily efforts of growing vegetables likewise created a moment of well-being in a patient. To this old homesick woman, the memory of her husband and their usual food moved her from her vulnerability as an “exiled,” isolated person (Galvin & Todres, 2013, p. 100) into a moment of dwelling, by telling her story about her husband and providing her with an important reason for recovery. Vegetables of a soft, familiar type, steamed or boiled, that she missed, were easy to chew and could have made her “feel at home” while being in the hospital.

The ability of the patients to receive the appropriate, prescribed medication was challenged by their inability to capture the tablet due to trembling, stiff or shaky hands, their impaired vision or a mouth too dry to swallow the medication. Dry mouth (Xerostomia) is most common due to systemic medications, which puts older patients at greater risk as they often consume more medication (Cassolato & Turnbull, 2003). These conditions are serious threats to the older patients’ health. Also, patients may experience bodily discomfort and pain if their medication is irregularly or incorrectly consumed (Galvin & Todres, 2013). Further, patients may experience a loss of identity when being unable to perform the procedure of medicine consumption themselves that challenges their well-being (Galvin & Todres, 2013).

A limitation of this study could arguably be the limited number of participants. However, the aim of qualitative research is not the measure but the depth of description. Data saturation is not a specific aim of ethnography; however, the data collection was ended when the first author judged that enough data was obtained and that additional participants would not add substantial new knowledge to the analysis. However, the researcher (LU) continuously sought to consciously reflect on the interaction of her pre-existing notions of the field and the practices observed. Furthermore, one strength of the study was the collaboration between the two researchers throughout the analysis, thereby ensuring a different reflection on the material produced. Through the data analysis, the dialogue led to insights and a deeper interpretation from the interaction of the two research positions.

The in-depth understanding of the situation of the vulnerable older patients brought out through the combination of participant observation and interviews is a significant strength of this study. As patients often tend to find it difficult to express their experience of clinical interactions for different reasons, the ability of this study to follow the life of a patient for a day in the hospital provided a unique potential for additional perspectives on experiences that patients may find difficult to verbalize. Older surgical patients make up a new group of patients as new surgical techniques and the developments in anesthesia have removed age as a limitation to surgery.

The description of the experiences of these older patients offered through this systematic data collection and data analysis may guide additional interventions of nursing care aiming at creating further insights and evidence in the area. Situation-oriented individual nursing care to enhance patients’ well-being during short-term admission to hospital is known to be a challenge (Bundgaard et al., 2012). During a short admission in a surgical ward, the challenge for both patients and staff is to be well prepared for this short meeting with focus on the individual patient and to be present in the situation communicating and using their senses.

## Conclusion

Older patients need to prepare themselves for hospital treatment and to obtain information about what may affect their well-being during their stay and how to prevent malnutrition. Focus on their oral cavity is, as we emphasize in this study, central to such preparation, because the mouth is the natural gateway for the additional nutrition and medication that these patients most commonly need following surgery. Their appetite may be significantly reduced and the patient information provided prior to surgery should deal with how to overcome this situation both short and long term. Polypharmacy of the older patients further adds to the challenge of sufficient care in this patient population. To make sure each tablet is grasped and swallowed may require additional support of individual routines, which can be highly time-consuming. This may pose a risk for such needs to be overlooked during a brief hospital stay. The perceptions, choices, needs, and consumption of food and drinks during illness and treatment should be assessed in each old patient, together with the status of their oral cavity and teeth. Such an assessment could be included in the first appointment the patient has prior to admission to the surgical ward.

Older patients seem to be on their own when it comes to individual challenges from their teeth or oral cavity, specific nutritional needs, or medication during surgical treatment for cancer. Short-term hospitalization requires a focused collaboration between staff and patient when it comes to the necessary consumption of food, drink, and medication.
